# Immunohistochemical Markers of Apoptotic and Hypoxic Damage Facilitate Evidence-Based Assessment in Pups with Neurological Disorders

**DOI:** 10.3390/vetsci8100203

**Published:** 2021-09-22

**Authors:** Ksenia Orekhova, Sandro Mazzariol, Beatrice Sussan, Massimo Bucci, Federico Bonsembiante, Ranieri Verin, Cinzia Centelleghe

**Affiliations:** 1 Department of Comparative Biomedicine and Food Science, University of Padova AGRIPOLIS, viale dell’Università 16, 35020 Legnaro, Italy; sandro.mazzariol@unipd.it (S.M.); federico.bonsembiante@unipd.it (F.B.); ranieri.verin@unipd.it (R.V.); cinzia.centelleghe@unipd.it (C.C.); 2 Department of Animal Medicine, Production and Health, University of Padova AGRIPOLIS, viale dell’Università 16, 35020 Legnaro, Italy; Beatrice.sussan@unipd.it (B.S.); Massimo.bucci@unipd.it (M.B.)

**Keywords:** seizures, hypoxic brain injury, canine diacylglycerolkinase zeta, beta-amyloid precursor protein, Apaf1 protein, Bcl-2 protein, MDR1 mutation

## Abstract

Seizures in puppies often present a diagnostic challenge in terms of identifying and treating the underlying cause. Dog breeds with mutations of the *MDR1*-gene are known to show adverse reactions to certain drugs, yet metabolic imbalance exacerbated by physiologically immature organs and other contributing pathologies require consideration before arriving at a diagnosis. This study analysed the brains of two male, 5-week-old Australian Shepherd siblings that died after displaying severe neurological symptoms upon administration of MilproVet^®^ to treat severe intestinal helminth infection. Despite the initial symptoms being similar, their case histories varied in terms of the symptom duration, access to supportive therapy and post-mortem interval. Histopathology and immunohistochemistry were used to obtain more information about the phase of the pathological processes in the brain, employing protein markers associated with acute hypoxic damage (β-amyloid precursor protein/APP) and apoptosis (diacylglycerolkinase-ζ/DGK-ζ, apoptotic protease activating factor 1/Apaf1, and B-cell lymphoma related protein 2/Bcl-2). The results seem to reflect the course of the animals’ clinical deterioration, implicating that the hypoxic damage to the brains was incompatible with life, and suggesting the usefulness of the mentioned immunohistochemical markers in clarifying the cause of death in animals with acute neurological deficits.

## 1. Introduction

Puppies with seizures are challenging veterinary patients, with ongoing post-natal organ maturation predisposing them to metabolic crises such as hypoglycemia [1] and inability to adequately metabolize certain drugs [2]. Clinical parameters vary individually and daily during development, and breed-associated *MDR1* status may contribute significantly to arising neurological deficits [3]. This fragile physiological balance limits therapeutic options, commonly resulting in death. However, integrative evidence-based assessment may help in understanding the underlying neuropathogenesis. Neurotoxicity is often associated with brain hypoxia, neurodegenerative and apoptotic changes [4]. In this study, immunohistochemistry (IHC) of cell stress-indicator proteins was employed to visualize biochemical imbalance prior to its morphological manifestation [5] in seizuring puppies, comparing them to control brains of three dogs of varying PMI intervals and without neurological symptoms at the time of death.

A litter (*n* = 8) of 5-week-old Australian Shepherd puppies presented severe neurological symptoms (videos thereof may be found in the Supplementary Materials) following deworming with one pill of Milprovet^®^ each (containing 2.5 mg milbemycin oxime/25 mg praziquantel): owners refer seizures, tremors and ataxia with manifestation of severe abdominal pain 12–24 h after its administration. One male (Puppy-1) died before arrival to the Veterinary Teaching Hospital of the University of Padova. A second male (Puppy-2) succumbed about an hour after initiation of supportive therapy. Their littermates recovered within 1.5 h of flow-by oxygen administration, intravenous ringer-lactate (4 mL/kg/h) and Intralipid^®^ 20% (1 mL/kg; Fresenius Kabi) infusion. Puppy-3 suffered two convulsive crises 3 h upon initiation of supportive care, recovering after repeated diazepam administration. Supplementary Table S1 summarizes the anamnestic, clinical and post-mortem findings in the Australian Shepherd puppies.

## 2. Materials and Methods

Post-mortem analyses were performed 24 h and 20 min after death in Puppy-1 and Puppy-2, respectively, and samples processed for routine histology and IHC with haematoxylin counterstain, using the same equipment and protocols as previously described [6], and antibody-specific positive, negative and blank controls. Formalin-fixed, paraffin-embedded archived brain cortex tissues from three control dogs without neurological symptoms (Control-1, -2, and -3) were cut and IHC-stained in the same way to assess for qualitative differences in the IHC-patterns dependent on three increasing post-mortem intervals (PMI). Pertinent demographic and forensic information on the control dogs is displayed in Table 1. Their *MDR1* phenotype is unknown.

Images were scanned with a D-sight scanning microscope at ×400 magnification (A. Menarini Diagnostics). A board-certified pathologist (R.V.) checked all the slides to assess histological and IHC findings.

Primary polyclonal antibodies used for IHC included anti-amyloid precursor protein (Abcam, #ab15272, diluted 1:50), rabbit anti-diacylglycerolkinase-ζ (DGK-ζ; MyBiosourse, #MBS2026991, 1:100), anti-apoptotic protease activating factor-1 (Apaf1; Enzo, #ADI-905-179-100, 1:500), and anti-B-cell lymphoma related protein (Bcl-2; Abcam, # ab196495, 1:150). The whole litter of Australian Shepherd puppies was tested for *MDR1*-gene mutation via allelic discrimination [7].

## 3. Results

Gross findings in the Australian Shepherd puppies were scant, with both livers appearing mildly enlarged, with fragile consistency, light-red coloration and rounded edges. Additionally, Puppy-2 displayed multifocal to coalescing pulmonary consolidation.

Histopathology confirmed the mild to moderate edema and congestion observed in all examined internal organs, and further revealed multifocal cortical laminar necrosis, superficial spongiosis and meningeal congestion of mild and mild to moderate severity in Puppy-2 and Puppy-1, respectively. Multifocal Purkinje cell degeneration was observed in the cerebellum, with perivascular edema of the molecular and Purkinje cell layers and the white matter (WM). These changes were mild and multifocal in Puppy-2, but moderate and multifocal to coalescing in Puppy-1, with additional Purkinje single cell necrosis. Both animals had mild to moderate, diffuse, micro- and macro-vesicular hepatic steatosis, and mild multifocal subacute lymphohistiocytic enteritis with reactive GALT. Both lungs displayed mild to moderate, multifocal, subacute, histiocytic interstitial pneumonia. Multifocal atelectasis, severe perivascular edema, interstitial hemorrhages, and a focal eosinophilic granuloma were observed in Puppy-2, while Puppy-1 presented mild emphysema at the lobe tip, multifocal interstitial and alveolar hemorrhages, and agonal edema. Heterozygous *MDR1*-gene mutations were present only in Puppy-1 and Puppy-4—the rest presented the wild-type phenotype.

**Figure 1:**
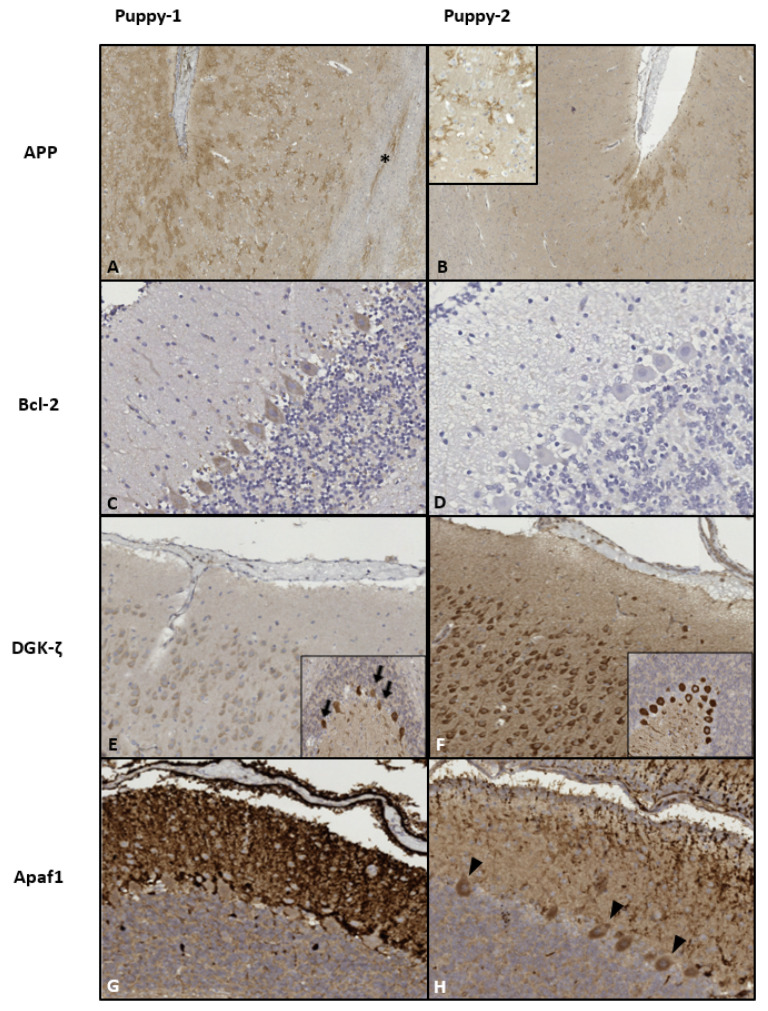
Immunohistochemicalpatterns of the implemented protein markers. (**A**,**B**): Neocortex. β-amyloid precursor protein/APP localized to neuronal synaptic membranes, multifocally enhanced against a light background in the superficial cortical layers. Intense staining was diffuse in Puppy-1, with affected white matteraxons (*). In Puppy-2, strong intensity was limited to the sulci; magnification ×50. Inset in (**B**) (×200): detail of arborized synaptic pattern. (**C**–**H**): apoptotic markers. (**C**,**D**): Moderate diffuse Purkinje cell cytoplasmic B-cell lymphoma related protein 2/Bcl-2-signal (with slight emphasis on gyral crowns; not shown) in Puppy-1 vs. predominantly negative cerebellum in Puppy-2. Magnification ×400. (**E**,**F**): Neocortex: “faded” appearance in Puppy-1 vs. strong cytoplasmic diacylglycerolkinase-ζ/DGK-ζ-signal in neurons and neuropil in Puppy-2; magnification ×100. Insets depict the puppies’ cerebella, whereby Puppy-1 displays more neuronal loss (arrows). In both animals, immunoreactivity was more pronounced in the gyral crowns, as opposed to the sulci (not shown). (**G**): Cerebellum of Puppy-1 shows intense and diffuse, predominantly axonal apoptotic protease activating factor 1/Apaf1 immunoreactivity in the molecular layer of cerebellar folia, while in Puppy-2, intensity is moderate and the pattern is multifocal to coalescing (**H**). The neuronal cytoplasm is negative in Puppy-1, yet there are multiple gyral foci of moderate immunoreactivity in Puppy-2 (arrowheads in (**H**)); Magnification ×200.

IHC results for the brain and the markers used are summarized in Table 2. In general, immunoreactivity was more intense and diffuse in Puppy-1. The specific IHC findings are depicted in Figure 1.

Control brain tissues were included for comparison to a dog that did not have any neurological symptoms at time of death (Control-1; PMI: 24 h), likely having endured only mild agonal hypoxia. Two control puppies presenting neonatal immaturity and additional inflammatory symptoms in the lungs (Control-2; PMI: 48 h) and metabolic imbalance as evidenced by perihepatic mineralizations (Control-3; PMI: 72 h) were included to assess potential changes of IHC-signal deriving from post-mortem protein degradation.

APP-reactivity of Control-1 was limited to a very mild, arborized or granular signal around single cortical neurons. Controls-2 and -3 both presented a background in the neuropil that was lighter than in both Australian Shepherd puppies, with multifocal cytoplasmic and synaptic neuronal immunoreactivity (+/++), with a mildly enhanced signal in the capillary vessel walls and meninges of Control-2. Control-2 and Control-3 were negative for Bcl-2, while Control-1, being mostly negative, presented findings more subtle, but reminiscent of those in Puppy-2, with a few foci of mild, cytoplasmic immunoreactivity (+) in neurons and neuropil. DGK-ζ was detected only in neuronal nuclei of Control-1 (+/++), while alternating between nuclear and cytoplasmic compartments in Control-2 (++/+++) and appearing exclusively in the cytoplasm in Control-3 (+). Apaf1-signal was most prominent as a radiating pattern from the superficial cortical layers and choroid plexus-adjoining regions in Control-2, and as a multifocal to coalescing immunoreactivity of endothelial cells and meninges (+++) in Control-3. No Apaf1 signal was observed in Control-1.

## 4. Discussion

Despite few macroscopic changes, histopathology revealed that the puppies were compromised under several aspects. Severe intestinal parasitic infection could have led to deficits in energy uptake and protein absorption in a challenging period such as weaning, inflicting initial damage to a physiologically immature liver [8]. Large breeds are more resistant to hypoglycemia than toy breeds [9], yet all dehydrated and anorectic puppies <4 months of age are highly susceptible [10]. While steatosis is a common finding in sudden pediatric death, it is not usually the sole cause [11]. Acute liver failure leads to hepatic encephalopathy displaying corticocerebral necrosis, edema and meningitis [12]. Due to the severity of parasitic infestation, additional complications of deworming, i.e., systemic resorption of parasitic endotoxin-like substances not only could have damaged the liver, but directly lead to a hypersensitivity reaction with symptoms similar to those of septic shock [13]. Systemic edema may have resulted from this, contributing to hypo-oxygenation of blood in the lungs. Mild to moderate interstitial pneumonia, subsequent to helminthic larval migration, likely impeded gas exchange further. In this case, evidence is insufficient to establish direct nematode-induced neurotoxicity.

Lesions caused by hypoxia were evident in the brains of both Australian Shepherd puppies. Cerebellar Purkinje cells are notoriously sensitive to acute hypoxic stress [14], and their degeneration may have explained some of the motor control deficits. Furthermore, IHC of the brains allowed a detailed look into the phase of neuronal degeneration, and a comparison with available controls without neurological findings.

APP is a synaptic membrane protein that plays a role in various physiological functions like axonal outgrowth and neuronal adhesion. It is expected in moderate concentrations in the developing brain [15]. Under pathological conditions like traumatic [16] and hypoxic-ischemic brain injury [5], APP may accumulate in damaged axons within minutes after exposure to the stressor [17]. No trauma pre-existed in this case. Therefore, we could reasonably state that the strong immunoreactivity of the dendritic synapses indicates acute hypoxic damage of cortical neurons and their axons in the WM. A more widespread distribution of the immunoreactivity in Puppy-1 suggests a more severe progression of the hypoxic-ischemic injury that led to its earlier death. Non-neurological controls presented overall lighter immunoreactivity, consistent with Control-1 experiencing the shortest duration of hypoxia coinciding with a short agonal period, comparing with Control-2 and -3 puppies that presented evidence of lung atelectasis, and therefore a longer ante-mortem hypoxic condition. Despite this, Controls-2 and -3 had a lighter and mostly background or vascular APP reactivity, and so differed to the stronger background and more pronounced synaptic and axonal reactivity of Puppy-1 and -2, implying that seizures induce a stronger degenerative and apoptotic stimulus in an acute timeframe compared to predominantly hypoxic conditions.

The DGK-ζ isoform physiologically localizes in the nucleus of neurons, but has been shown to acutely and irreversibly translocate into the cytoplasm of hippocampal neurons following ischemic injury, before gradually disappearing [18]. Its absence putatively indicates a “point-of-no-return”, anticipating apoptosis [19]. The intense immunoreactivity of the neuronal cytoplasm in Puppy-2 could match the acute respiratory distress leading to hypoxia and death, as mirrored by Control-2 and to a lesser extent, Control-1, while the faded appearance of Puppy-1’s cortex may reflect a later phase of widespread neurodegeneration incompatible with life. Furthermore, Control-1 displayed exclusively intranuclear neuronal DGK-ζ, implying a largely physiological neurochemical profile affected mildly by the agonal process.

Bcl-2 is an anti-apoptotic protein [20], yet according to Shinoura and colleagues [21], it can be pro-apoptotic in higher concentrations. Its multifocal appearance in Puppy-1’s neuronal cytoplasm in the context of a lack of nuclear DGK-ζ -expression and overlapping with Apaf1-immunoreactive neurons of the gyri could therefore imply a pro-apoptotic or ineffective compensatory mechanism preceding widespread neuronal apoptosis. Lack of Bcl-2-immunoreactivity in Puppy-2’s neurons could suggest the predominance of acute, pro-apoptotic mechanisms, with a milder expression in astrocytes highlighting their greater resilience to hypoxia compared to neurons. Controls were mostly negative apart from the focal mild granular neuropil reactivity in Control-1 (displaying similarity to Puppy-2), thereby implying that Bcl-2 protein upregulation as visualized by the herein employed IHC-antibody is detectable in animals that experienced a comparatively stronger apoptotic stimulus surpassing that expected during acute agonal hypoxia, such as in the seizuring Puppy-1.

Apaf1 reflects intrinsic apoptosis upstream of executioner caspase-activating caspase 9 [22]. Multifocal to coalescing axonal and WM-glial immunoreactivity in Puppy-1 may therefore indicate an advanced stage of neurodegeneration, since glia generally succumb to hypoxia later than neurons [23]. A similar pattern was observed in the Control-2 and -3 puppies, with Control-2 presenting the most disseminated axonal granular immunoreactivity, and an additional multifocal to coalescing endothelial signal, potentially indicative of vascoconstriction-induced apoptosis [24]. It represents evidence that early apoptotic mechanisms, next to acute necrosis observed in the hematoxylin-eosin sections, played a role in the neuronal degeneration of both the control and the Australian Shepherd puppies, but not in the adult Control-1.

Relevant IHC-findings in Puppy-1 include widespread APP-, faded DGK-ζ- and multifocal Bcl-2-immunoreactivity in cortical neurons simultaneous to necrotic shrinkage, diffuse cerebellar early-apoptotic Apaf1-reactivity and severe necrosis as evidenced by complete multifocal disintegration of Purkinje-neurons. This pattern matches advanced necrotic and progressive apoptotic changes. Puppy-2’s central nervous system rather reflects acute necrosis and pre- to early apoptosis with intense cortical DGK-ζ-, multifocal cerebellar Apaf1- and lack of Bcl-2-expression, and multifocally shrunken, but mostly intact neurons. Though apoptosis is more prominent in immature brains if compared to adults [25], necrotic emphasis putatively indicates more severe hypoxic-ischemic insults, with delayed apoptosis, by itself, associated with milder damage [26]. Thus, Puppy-1 conceivably experienced more severe and longer-lasting brain injury than Puppy-2. Compared with available control puppies with evidence of severe ante-mortem hypoxia, the findings in Puppy-1 in particular, and Puppy-2 to a lesser extent, indicate that a slightly different, or stronger noxious stimulus led to the neurochemical imbalance visualized by the employed IHC markers in the Australian Shepherd siblings. The hepatoencephalic toxicity exacerbated by parasitic die-off induced by macrocyclic lactone administration could have represented such a stimulus. The stronger differences between all the puppies and the adult Control-1 with no neuropathological findings, especially in the case of the intranuclear vs cytoplasmic DGK-ζ distribution and intensity, and the low APP and lack of Bcl-2 and Apaf1 signal, appear to underline this.

Single reports describe dogs with heterozygous *MDR1*-gene mutations responding to macrocyclic lactones with mild depression and ataxia, or more severe, transient (<48 h) neurotoxicosis preceding full recovery [27]. Furthermore, milbemycin oxime at dosages < 2.5 mg/kg is postulated as a safe macrocyclic lactone, even in sensitive dogs [28]. Compromised by dyspnea and an injured liver, however, even a mildly reduced function of the blood-brain-barrier P-gp channel protein, responsible for the efflux of macrocyclic lactones from the brain cells to prevent neurotoxicity [29,30], may have contributed to the faster progression of brain injury and earlier death in Puppy-1. Atelectasis and severe perivascular edema probably triggered the terminal respiratory distress in Puppy-2, resulting in more acute hypoxia-induced necrosis and apoptosis.

Puppy-4, also being a heterozygous *MDR1* mutant, responded well to the supportive care. Meanwhile, *MDR1*-wild-type Puppy-3 suffered further seizures but responded well to diazepam, unlike dogs with moxidectin toxicity [31]. These facts underline the significant contribution of severe metabolic imbalance and parasite-associated dyspnea to the two fatalities.

This study is based on diagnostic cases, and therefore contains inevitable limitations. In particular, the selection of control animals without neurological changes is problematic, thus our knowledge on a healthy profile for the dog brain is limited. Ideally, controls would be healthy experimental, age-, sex-, and breed-matched dogs that had little to no hypoxic changes within the brain tissues at the time of death. In reality, every animal, whether dying of natural causes or euthanasia, experiences some extent of agonal hypoxia [32], therefore some overlap between the results of Puppies-1 and -2 and the controls is not surprising. In our case, the controls had to be selected from archived tissues from animals submitted to the University Veterinary Necropsy Service, thus limiting the possibility of an age-matched control without respiratory distress. Controls-2 and -3 were more appropriate in age, but some vascular changes were noticed. Therefore, they could only serve as a preliminary comparison for the confounder of a longer PMI.

In this respect, consistency between clinical state and IHC findings, as well as similar IHC-patterns seen in animals with a PMI of up to about 72 h, render it unlikely that the longer post-mortem interval of Puppy-1 significantly compromised IHC results. The robustness of some protein markers in human post-mortem neuronal tissue is postulated to be conserved at a PMI of up to 50 h, though this varies by marker [33]. Changes in Bcl-2-expression have previously been suggested as reliable markers correlating negatively to increasing PMI [34], yet in our case, Puppy-2 with the shortest PMI of 20 min displayed the least Bcl-2-expression, while dogs with a PMI of 24 h (Puppy- and Control-1) yielded a slightly stronger IHC signal. Thus, further research employing a larger number of experimental animals is required before asserting a reliable PMI threshold for IHC evaluation in canines. Nevertheless, results obtained in the context of this diagnostic case suggest that above-mentioned IHC markers could merit systematic validation using orthogonal proteomic methods such as Western blot [35].

## 5. Conclusions

Considered together, the anamnestic, clinical, histological and immunohistochemical findings point to a multifactorial hypoxic syndrome as the cause of acute neuronal degeneration, contributing significantly to the disruption of vital functions and resulting in the puppies’ deaths. Direct drug toxicity is unlikely but cannot be excluded, since single-gene approaches to pharmacogenetics leave considerable individual variation to be explored with pharmacogenomics [36]. The recovery of 6/8 littermates with supportive care emphasizes the greater likelihood of a metabolic imbalance and liver damage exacerbated by parasitic die-off. Evaluation of IHC markers in the context of the case helped interpret severity and pathogenesis of brain lesions before morphological changes became evident, implying that while general neurodegenerative and apoptotic processes are common, the nature of the noxious stimulus may allow neurochemical differentiation between agonal or perinatal hypoxia and seizures owing to (e.g., hepatoencephalic) toxicity. To our knowledge, this is the first report of nucleocytoplasmic translocation of DGK-ζ in cortical neurons. While results are preliminary and the influence of PMI on protein expression needs further research, they demonstrate that appropriate validation of IHC-markers holds great potential as an ancillary forensic tool for patients presenting neurological deficits without a clear etiology.

## Figures and Tables

**Table 1:** Pertinent demographic and forensic information on the five dog brains included in this study. M: male; F: female.

Control Dog (M/F)	Breed	Age	Cause of Death	Pertinent Pathological Findings	Post-Mortem Interval
Puppy-1 (M)	Australian Shepherd	5 weeks	To be determined	Hepatic, enteric, respiratory and central nervous system damage (described in “Results”)	24 h
Puppy-2 (M)	Australian Shepherd	5 weeks	To be determined	Hepatic, enteric, respiratory and central nervous system damage (describe in “Results”)	20 min
Control-1 (F)	Toy Poodle	12 years	Cardiovascular insufficiency	Aortic aneurism; monolateral inner ear infection	24 h
Control-2 (F)	Shitzu	1 week	Neonatal immaturity and pneumonia potentially resulting in septicemic shock	Diffuse atelectasis; diffuse severe pleuropneumonia; moderate multifocal tubular necrosis, mild diffuse gastroenteritis	48 h
Control-3 (M)	Dogue de Bordeaux	1 week	Neonatal immaturity and cardiorespiratory insufficiency	Diffuse atelectasis; ascites (serosanguinous); perihepatic vascular mineralisations	72 h

**Table 2:** Summary of qualitative immunohistochemical assessment of the neocortices and cerebella of the two tested puppies. Immunoreactivity with **−/+**: very light; **+**: light; **++**: moderate and **+++**: strong intensity. **-**: no immunoreactivity. PMI: post-mortem interval. WM: white matter.

Antibody	Puppy	Puppy-1			Puppy-2		
	PMI	24 h			20 min		
	Cell Type	Neurons	Glia	Meninges/ Endothelia	Neurons	Glia	Meninges/ Endothelia
**APP**	Cortex	**+++** Synaptic membranes Diffuse	**++** Light background + Multifocal coalescing axons in WM	**-**	**++** Synaptic membranes in sulci	**+** Light background	**-**
	Cerebellum	**++** Cytoplasmic	**+** Light background	**-**	**+++** Cytoplasmic	**++** Light-moderate background	**-**
**Bcl-2**	Cortex	**+++** Cytoplasmic Multifocal in gyri	**+/++** Granular Diffuse	**+++** Leptomeninx Multifocal **++** Cytoplasmic, choroid plexus endothelia Diffuse	**-**	**+** Granular Diffuse, mild pericapillary emphasis	**++** Leptomeninx Few foci
	Cerebellum	**++** Cytoplasmic Multifocal in gyri	**-**	**-**	**−/+** very light cytoplasm, Single foci	**-**	**-**
**DGK-ζ**	Cortex	**+** Cytoplasmic Diffuse	**+** Light background **++** Astrocyte cytoplasm Multifocal	**+** Leptomeninx Multifocal	**+++** Cytoplasmic Diffuse	**++** Moderate background **+++** Astrocyte nuclear Multifocal	**+++** Endothelia Diffuse
	Cerebellum	**++/+++** Cytoplasmic Diffuse, with multifocal shrunken, deformed cells	**+**	**−/+** Leptomeninx Single foci	**+++** Cytoplasmic, Diffuse; Nuclear, Multifocal	**−/+**	**++** Endothelial Multifocal
**Apaf1**	Cortex	**-**	**+++** Cytoplasmic and nuclear Multifocal in WM	**+++** Leptomeninx Multifocal	**-**	**-**	**++** Small/mid-caliber vessels Multifocal
	Cerebellum	**-** Cytoplasmic **+++** Diffuse in molecular layer axons	**+** Light background	**+++** Leptomeninx Diffuse	**++** cytoplasmic Multifocal in gyri **++/+++** Multifocal-coalescing in sulci	**+** Light background	**+/++** Leptomeninx Multifocal-coalescing

## Supplementary Materials

The following are available online at www.mdpi.com/article/10.3390/vetsci8100203/s1, Table S1: Case summary for the two Australian Shepherd puppies that died following Milprovet^®^ administration, Video S1: Neurological symptoms of Puppy-1 (~12 h after Milprovet^®^ administration), Video S2: Neurological symptoms of Puppy-1 (~14 h after Milprovet^®^ administration). The videos show Puppy-1 is displaying apathy, ataxia, tremors, incoordination and dyspnea that all got progressively worse over a period of several hours prior to a comatose state followed by death.
