# Supplementary material for: Immunohistochemical Markers of Apoptotic and Hypoxic Damage Facilitate Evidence-Based Assessment in Pups with Neurological Disorders
Source: Vet Sci. 2021 Sep 22;8(10):203. doi: 10.3390/vetsci8100203 (PMC8537515; doi:10.3390/vetsci8100203)
Supplement: Supplementary file 1 [file vetsci-08-00203-s001.zip › Supplementary_TableS1.pdf]

<table>
<tr><th>Findings</th><th>Puppy-1 (Male)</th><th>Puppy-2 (Male)</th></tr>
<tr><td rowspan="3">Anamnestic</td><td>Heterozygous MDR1-gene mutation (along with one other puppy – Puppy-4 – in litter; n=8)</td><td>Normal MDR1 phenotype</td></tr>
<tr><td colspan="2">Whole litter received Milprovet® (2.5 mg milbemycin oxime/25 mg praziquantel) to treat severe intestinal parasitic infection
12-24 hrs after Milprovet® administration, symptoms appear and worsen:
- Seizures
- Tremors
- Ataxia</td></tr>
<tr><td>Dies en route to veterinary hospital</td><td></td></tr>
<tr><td rowspan="2">Clinical</td><td colspan="2">Above-mentioned symptoms persist (40 hrs after Milprovet®). Additionally:
- depression with hyper-excitability toward any external stimuli
- Hyperthermia: 39-40 °C rectal temperature
- Tachycardia
- Mild dyspnea and tachypnea with homogeneous increase in vesicular sounds
Littermates, including one other heterozygous MDR1-mutant, recover within 1.5 hrs of supportive therapy: flow-by oxygen, i.v. ringer-lactate (4 ml/kg/hr) and Intralipid®20% (1ml/kg) administration. 1 littermate (Puppy-3, with homozygous MDR1 wild type phenotype, has two convulsive crises 3 hrs after initiation of therapy, but recovers after repeated diazepam administration (1st dose endorectal, 2nd dose intravenous).</td></tr>
<tr><td></td><td>Dies 1hr after initiation of supportive therapy</td></tr>
<tr><td rowspan="3">Gross Pathology</td><td>Post mortem interval (PMI): 24 hr</td><td>PMI: 20 mins</td></tr>
<tr><td colspan="2">Both livers appearing mildly enlarged, with fragile consistency, light-red coloration and rounded edges</td></tr>
<tr><td></td><td>multifocal to coalescing pulmonary consolidation</td></tr>
<tr><td>Microscopic</td><td colspan="2">Mild to moderate edema and congestion in all examined internal organs</td></tr>
</table>

pathology	Cerebrum: cortical laminar necrosis, superficial spongiosis and meningeal congestion	
	mild to moderate	mild
	Cerebellum: multifocal Purkinje cell degeneration with perivascular edema of the molecular and Purkinje cell layers and the white matter	
	mild to moderate, coalescing, with multifocal Purkinje cell necrosis	mild
	Liver: mild to moderate, diffuse, micro- and macro-vesicular hepatic steatosis	
	Intestine: mild multifocal subacute lymphohistiocytic enteritis with reactive GALT	
	Lungs: mild to moderate, multifocal, subacute, histiocytic interstitial pneumonia.	
	mild emphysema at the lobe tip, multifocal interstitial and alveolar hemorrhages, and agonal edema	multifocal atelectasis, severe perivascular edema, interstitial hemorrhages, and a focal eosinophilic granuloma

Supplementary Table S1: Case summary for the two Australian Shepherd puppies that died following Milprovet® administration.
